# High Proteolytic and Collagenolytic Activity in an Environmental *Vibrio* Isolate: Insights into Tissue-Degrading Virulence Factors

**DOI:** 10.3390/ijms262010153

**Published:** 2025-10-18

**Authors:** Monica Salamone, Aldo Nicosia, Giulio Ghersi, Angela Cuttitta, Paola Quatrini, Marcello Tagliavia

**Affiliations:** 1 Institute for Biomedical Research and Innovation, Italian National Research Council (IRIB-CNR), Via Ugo La Malfa, 153, 90146 Palermo, Italy; monica.salamone@irib.cnr.it (M.S.); aldo.nicosia@irib.cnr.it (A.N.); 2 Department of Biological, Chemical and Pharmaceutical Sciences and Technologies (STEBICEF), University of Palermo, Viale delle Scienze, Ed. 16, 90128 Palermo, Italy; giulio.ghersi@unipa.it; 3 Institute for the Study of Anthropic Impact and Sustainability in the Marine Environment, Italian National Research Council (IAS-CNR), Via del Mare, 3 Torretta Granitola, 91021 Campobello di Mazara, Italy; 4 Institute for Mediterranean Studies, Italian National Research Council (ISMed-CNR), Via Filippo Parlatore, 65, 90145 Palermo, Italy; 5 Department for Earth and Marine Sciences, University of Palermo, Via Archirafi, 36, 90128 Palermo, Italy; paola.quatrini@unipa.it

**Keywords:** *Vibrio*, proteases, virulence factors, collagenases

## Abstract

*Vibrio* is a genus of ubiquitous aquatic bacteria that includes numerous pathogenic species. Their remarkable genomic plasticity and rapid evolution make them of particular interest from both clinical and ecological perspectives. Successful infection by *Vibrio* species often relies on multiple virulence factors, including secreted enzymes. Here, we report the characterization of a novel environmental *Vibrio* strain isolated from a wild octopus that developed fulminant septicaemia accompanied by widespread soft tissue lysis. These severe symptoms prompted a detailed investigation into the bacterium’s identity and enzymatic profile, focused on proteases as potential virulence factors. Multi-locus sequence analysis placed the isolate within the Harveyi clade but revealed no perfect match to known strains, supporting its designation as a novel strain. Biochemical assays demonstrated strong proteolytic—including collagenolytic—activity, which makes this strain a promising source of enzymes for biotechnological applications.

## 1. Introduction

*Vibrio* is a genus of rod-shaped, Gram-negative γ-proteobacteria inhabiting diverse aquatic ecosystems, including marine, estuarine, and brackish waters [1,2]. These bacteria exhibit remarkable ecological versatility and genomic adaptability, supported by a unique genomic architecture comprising two circular chromosomes. Chromosome I typically encodes essential metabolic and housekeeping genes, whereas chromosome II—believed to have evolved from an ancestral megaplasmid—contains genes linked to niche adaptation, environmental sensing, and virulence [3,4].

The genomes of *Vibrio* species are highly dynamic, undergoing frequent structural rearrangements driven by high rates of horizontal gene transfer (HGT), homologous recombination, and the activity of mobile genetic elements such as transposons, integrons, prophages, plasmids, and genomic islands [5,6,7]. A distinctive feature in several species, most extensively studied in *V. cholerae*, is the super-integron, a large genetic platform capable of capturing diverse gene cassettes, which facilitates the acquisition of novel functions, thereby accelerating adaptation and evolution [7]. This genomic plasticity allows *Vibrio* species to rapidly respond to environmental change, colonize novel hosts, and acquire new functional traits, including an expanded enzymatic repertoire [5,6].

Over 100 *Vibrio* species have been described, with at least 12 recognized as human pathogens [8]. The most studied, *V. cholerae*, is the etiological agent of cholera, a severe diarrheal disease transmitted via contaminated water. Other clinically relevant species include *V. parahaemolyticus*, *V. alginolyticus*, and *V. vulnificus*, which typically cause vibriosis through ingestion of contaminated seafood or exposure to seawater, particularly in immunocompromised individuals [1,9]. The global incidence of vibriosis is rising, partly due to climate change and associated increases in sea surface temperatures, which are thought to promote proliferation, geographic expansion, and possibly enhanced virulence in *Vibrio* populations [2,10,11,12,13,14].

Beyond human health, various *Vibrio* species are significant pathogens in aquaculture and marine wildlife, causing recurrent outbreaks in vertebrate and invertebrate hosts. Species such as *V. anguillarum*, *V. salmonicida*, *V. harveyi*, *V. coralliilyticus*, and *V. crassostreae*—most within the Harveyi clade—are associated with diseases in fish, mollusks, and crustaceans, leading to substantial economic losses [11,14].

*Vibrio* pathogenicity is multifactorial, involving an array of extracellular virulence factors, including hemolysins, enterotoxins, phospholipases, cytotoxins, proteases (such as collagenases and elastases), siderophores, and hemagglutinins; bacterial motility has also been implicated in virulence [15,16,17,18]. Among these factors, proteases play a pivotal role in bacterial invasiveness. *Vibrio*’s protease repertoire includes zinc metalloproteases (e.g., vibriolysin), serine proteases, cysteine proteases, and neutral elastase-like enzymes, all of which contribute to tissue degradation and invasion, immune evasion, and nutrient acquisition [12]. For example, vibriolysin degrades structural proteins such as collagen, elastin, and fibrinogen, facilitating tissue penetration and inflammatory responses [19]. In fish pathogens like *V. anguillarum*, proteases are essential for host colonization and mortality [20].

The production of virulence-associated proteases is generally regulated by environmental cues such as iron limitation, temperature, pH, and host-derived signals (e.g., bile salts, tissue contact) [21]. This regulation has been well documented in human pathogens like *V. cholerae* and *V. vulnificus* [21,22,23]. Nevertheless, many *Vibrio* proteases are also expressed under non-host conditions, albeit in a regulated manner, including in natural waters and laboratory culture media [21,22]. Notably, many of these enzymes are halo- and thermos-tolerant, and active across broad pH ranges, supporting roles in general survival strategies such as nutrient utilization and interspecies competition [23,24], in addition to host infection.

Biochemical versatility and diversity of proteases from *Vibrio* spp. make these bacteria an attractive source of molecules for biotechnological applications spanning medicine (e.g., tissue dissociation for regenerative medicine, wound treatment, etc.), food processing, industrial processes, and environmental remediation [17,25]. Importantly, their high genomic plasticity fosters the emergence of novel strains with unique combinations of enzymatic and pathogenic traits, rendering any isolate a potential source of new bioactive molecules.

Here, we characterize the extracellular proteolytic activities, potentially serving as virulence factors, of a novel *Vibrio* sp. isolated from a diseased wild octopus. The symptoms made us raise the hypothesis of a major role for proteases in the disease process, prompting molecular identification of the bacterium and detailed enzymatic profiling. The results support classification of the isolate as a strain within the *Harveyi* clade and reveal distinctive biochemical traits with potential relevance for microbial ecology, pathogenicity, and biotechnological applications, although direct experimental confirmation of its virulence was not undertaken.

## 2. Results and Discussion

### 2.1. Bacterial Isolation, Analyses of Enzymes Secreted from Vibrio Isolates and Phylogenetic Analyses

A severely injured wild octopus (*Octopus vulgaris*), likely attacked by a predator shortly before capture, was found to exhibit symptoms characterized by diffuse, rapidly progressing tissue swelling, disintegration, and necrosis. To investigate whether the pathology was associated with bacterial infection, hemolymph and affected tissues were aseptically collected and cultured on multiple media.

Bacterial loads of approximately 10^5^ cfu/g were detected in both hemolymph and tissues, consistent with systemic infection and peripheral tissue invasion, likely originating from the initial wound.

A preliminary screening aiming to identify putative vibrios was based on growth on thiosulfate–citrate–bile salts–sucrose (TCBS) agar and on PCR amplification using *Vibrio*-specific primers targeting the *rpoD* gene [26], which assigned most isolates to the genus *Vibrio*. 

**Figure 1:**
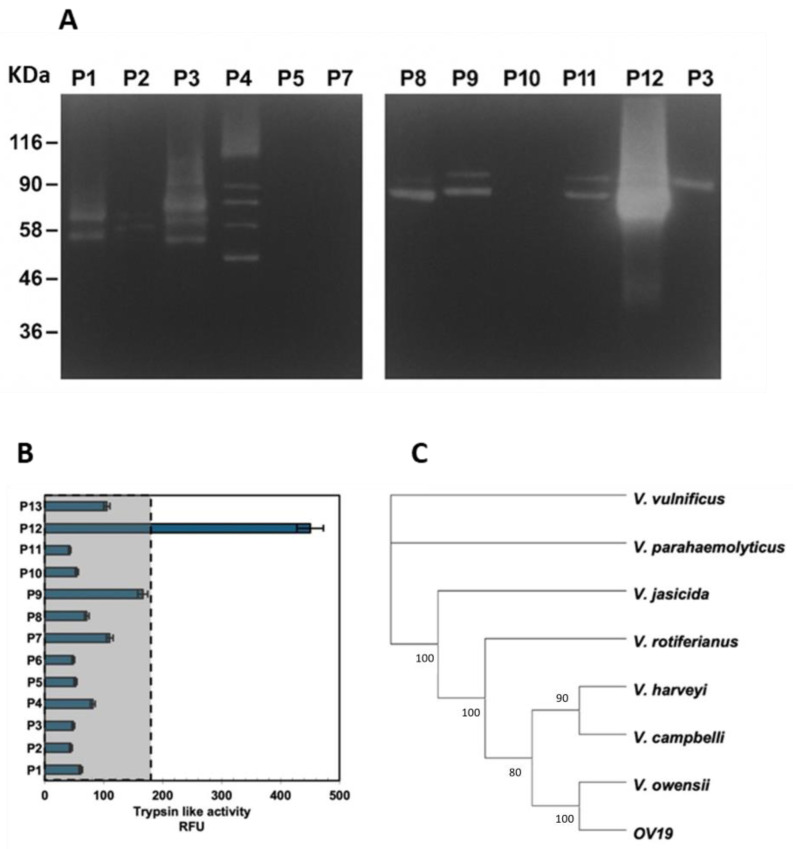
Enzymatic activities and phylogenetic relationships of *Vibrio* isolates. (**A**) Zymographic analysis of gelatinase activity of secreted proteases from liquid cultures of 13 *Vibrio* isolates (P1–P13). Molecular weight marker bands are reported. (**B**) Trypsin-like activity in supernatants of bacterial cultures. The dot line marks the maximum level of trypsin-like activity recorded in isolates other than OV19 (P12). Values are the average of three independent experiments. (**C**) Phylogenetic tree based on MLSA. The analysis was based on *ftsZ*, *gapA*, *gyrB*, *mreB*, *recA*, *rpoA*, and *topA*, *16S* rRNA concatenated sequences from members of the Harveyi clade and the novel isolate OV19 (profile P12). The evolutionary relationships were inferred using the maximum likelihood method and Tamura–Nei model. The bootstrap consensus tree inferred from 500 replicates is taken to represent the evolutionary history of the taxa analyzed. Branches corresponding to partitions reproduced in less than 50% bootstrap replicates are shown collapsed. The percentage of replicate trees in which the associated taxa clustered together in the bootstrap test are shown next to the branches. Evolutionary analyses were conducted in MEGA11.

The rapid tissue degradation and the absence of hemolymph clotting, suggestive of pathogenic mechanisms potentially involving potent degradative enzymes, prompted further characterization of the isolates focusing on their proteolytic potential. Sixty-five isolates were randomly selected from Marine Agar plates—including colonies representative of different phenotypes—for proteolytic activity screening. Secreted proteases from liquid cultures were analyzed by gelatin zymography, revealing 13 distinct exoprotease profiles. One profile, corresponding to isolate OV19 (sample P12; Figure 1), displayed particularly strong proteolytic activity compared with the others, and was selected for detailed biochemical characterization.

To compare quantitatively the proteolytic capacity of OV19 with that of representative isolates from each exoprotease profile group (P1–P13), one isolate per group was selected, and the trypsin-like activity in culture supernatants was measured using the specific fluorescent substrate BOC-Gln-Ala-Arg-AMC (Figure 1B).

OV19 exhibited the highest trypsin-like specific activity, with enzyme activity levels nearly fivefold greater than the mean of all other tested isolates. This marked proteolytic capacity reinforced the hypothesis that OV19 could possess higher tissue-destructive potential compared to other isolates, justifying in-depth investigations into its biochemical properties and pathogenic traits.

### 2.2. Molecular Identification of OV19

Given OV19’s distinctive proteolytic profile, molecular analyses were carried out to enable targeted comparative studies. Since *16S* rRNA gene sequencing alone is insufficiently discriminative for *Vibrio* taxonomy, analyses aiming at identifying the isolate at the species level were carried out firstly based on sequence analysis of *pyrH* [27], which assigned the isolate to *V. owensii*. A further analysis, based on *rpoA*, *recA* and *pyrH* sequences considered collectively [28] by BLAST analysis (BLAST+ 2.16.0), showed sequence identity exceeding 99.6% (99.8, 99.6, and 99.7%, respectively) with *V. owensii*; these percentages were all above the thresholds recommended to assign strains to the same species [28]. To achieve a more robust identification, multilocus sequence analysis (MLSA) was performed on concatenated sequences from seven protein-coding genes (*ftsZ*, *gapA*, *gyrB*, *mreB*, *recA*, *rpoA*, and *topA*) together with the *16S* rRNA gene, using the pipeline described in [29,30].

Each concatenated sequence was first subjected to PSI-BLAST to identify the closest *Vibrio* matches within a range of 95–100% sequence identity. The resulting dataset was then compared with six Harveyi clade species showing the highest sequence similarity (*V. campbellii*, *V. harveyi*, *V. rotiferianus*, *V. jasicida*, *V. owensii*, and *V. parahaemolyticus*) with *V. vulnificus* used as an outgroup. Neighbor-joining (NJ) and maximum-parsimony (MP) phylogenetic analyses produced congruent topologies, with *V. vulnificus* rooting the tree (Figure 1C).

The MLSA placed OV19 within the Harveyi clade, in close association with *V. owensii*, and at similar pairwise distances to *V. campbellii* (0.25) and *V. harveyi* (0.26). Sequence identity analysis revealed values of 98% for *ftsZ*, 97.4% for *gapA*, and 100% for *rpoA* relative to *V. harveyi*; 99.5% for *recA* and 97% for *mreB* relative to *V. owensii*; and 98% for *gyrB* and 99% for *topA* relative to *V. campbellii*. Therefore, the phylogenetic placement of OV19 appeared intermediate among these closely related type strains, suggesting a mosaic genomic composition. These data partially challenged the earlier identification of *V. owensii* based on either one or three *loci*, thereby underscoring the inherent limitations of current molecular approaches for *Vibrio* identification.

From an evolutionary perspective, these findings highlight the complexity and adaptability of the *Vibrio* genus, in which frequent horizontal gene transfer facilitates the acquisition of novel genetic material. Such processes may also contribute to shaping the enzymatic arsenal of *Vibrio*, promoting the emergence of new functional traits, including pathogenic ones [31,32]. Taken together, our data indicate that OV19 might represent a genetically distinct lineage—closely related to *V. owensii*—within the Harveyi clade. Further information on its genomic structure and functions might be achieved through whole-genome sequencing (WGS) and also from the perspective of biotechnological exploitation of specific genes. Moreover, WGS might help in obtaining insights into the emergence of such a strain, while allowing for a more precise taxonomic resolution. However, it is worth pointing out that taxonomic classification of vibrios is challenging due to their high genomic plasticity and recombination rates. Moreover, the robustness of any bioinformatic analysis strongly depends on the sequence’s availability in GenBank and on criteria employed to assign them at the species level, which is one of the major challenges in *Vibrio* taxonomy [33].

### 2.3. Comparison of Collagenolytic Activity of OV19 with Vibrio Type-Strains

To further profile the proteolytic activities of OV19’s secreted enzymes, we focused our analyses on collagenases, since the collagenolytic activity is a common feature of *Vibrio* strains, particularly of pathogenic and invasive ones [34]. We firstly compared the gelatinolytic activity of OV19 with that of *V. vulnificus* (well known for its collagenolytic activity) and several Harveyi clade members (*V. parahaemolyticus*, *V. campbellii*, *V. harveyi*, and *V. splendidus*). Supernatants from overnight cultures grown in appropriate liquid medium were collected, and their gelatinolytic activity was examined by gelatin zymography.

**Figure 2:**
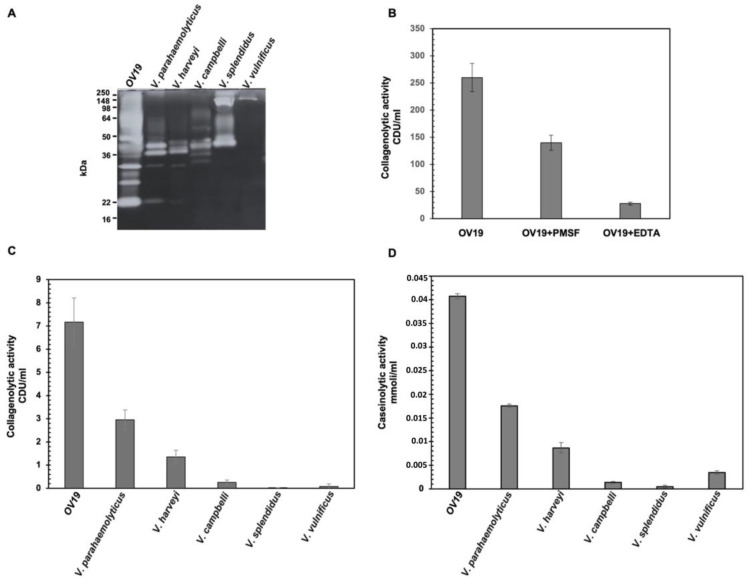
(**A**) Zymographic analysis of gelatinase activity of secreted proteases from OV19, *V. parahaemolyticus*, *V. campbelli*, *V. harveyi*, *V. vulnificus*, *V. splendidus*. On the left, positions of molecular weight marker bands are reported. (**B**) Inhibitory effects of PMFS and EDTA on the collagenolytic activity of OV19. (**C**) Collagenolytic activity in culture media of different *Vibrio*. The different vibrios were grown overnight in Marine Broth and the collagenase activity measured by colorimetric test using synthetic peptide Carbobenzoxy-Gly-Pro-GlyGly-Pro-Ala-OH. Collagenolytic activity was normalized with cell density. (**D**) Caseinolytic activity in different *Vibrio* strains. Bacteria were grown overnight in Marine Broth and the proteases activity measured by colorimetric test, using casein as substrate. Proteolytic activity was normalized with cell density. Data are the average of three independent experiments.

This comparative approach revealed marked differences in the proteolytic profiles of OV19 and the reference type strains (Figure 2A). The tested *Vibrio* strains produced enzymes spanning an apparent molecular mass range of ~20 to 150 kDa, with band relative intensity varying among species. Notably, the OV19 supernatant displayed a more complex pattern, with numerous bands across different molecular weights, indicative of a diverse and abundant extracellular protease repertoire.

To identify the major classes of proteases contributing to OV19’s activity, zymograms were performed in the presence of either EDTA or PMSF as inhibitors (Figure 2B). PMSF treatment reduced collagenolytic activity by approximately 50%, whereas EDTA inhibited it by more than 90%. These findings suggest that the majority of collagen-digesting proteases in OV19 are cation-dependent metalloproteases, with a significant fraction of activity also attributable to serine and/or cysteine proteases.

To further dissect the contribution of collagenases relative to other proteases, supernatants were tested for their ability to hydrolyze the specific collagenase substrate carbobenzoxy-Gly-Pro-Gly-Gly-Pro-Ala-OH alongside casein to monitor general proteolytic activity. Both assays confirmed that OV19 possesses strong collagenolytic and caseinolytic activities, both outperforming those of reference strains (Figure 2C,D).

Because the assays described above did not strictly distinguish between gelatinolytic and collagenolytic activities, we next investigated whether OV19 could degrade native, insoluble collagen, a defining feature of true collagenases capable of hydrolyzing triple-helix collagen. Such activity is common among invasive *Vibrio* strains and is known to facilitate tissue dissociation, bacterial invasion, and systemic dissemination [34].

To test this, OV19 and selected reference strains (namely *V. vulnificus*, *V. parahaemolyticus*, *V. harveyi*, *V. campbellii*, and *V. splendidus*) were individually inoculated into artificial seawater supplemented with insoluble bovine tendon collagen as the sole carbon and energy source. In this nutrient-limited medium, growth was expected to occur only in strains capable of effective collagen hydrolysis, most likely via secreted collagenases.

**Figure 3:**
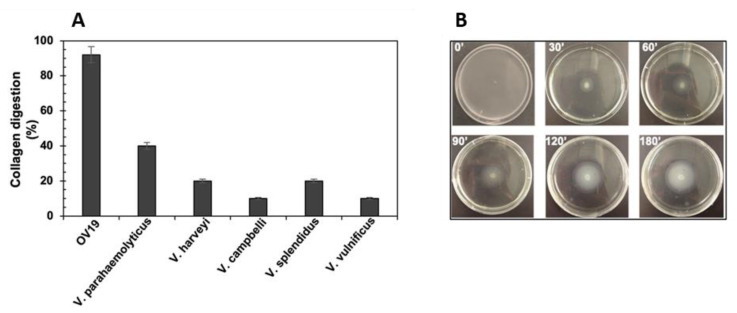
Pathogenic features of OV19. (**A**). Collagen hydrolysis in various *Vibrio* type-strains and OV19. (**B**) Swarming motility test of OV19 on Marine Agar plates, monitored over 3 h.

Over a 48 h monitoring period, OV19 exhibited clear growth and collagen solubilization, with visible bacterial proliferation emerging after approximately 30 h (Figure 3A). The extended lag phase may reflect the low initial inoculum density (10^5^ cfu/mL) and the requirement for gradual protease accumulation before sufficient soluble peptides could support growth. Once established, bacterial multiplication was presumably accompanied by sustained enzyme secretion, accelerating collagen degradation. The process was likely enhanced by the combined activity of collagenases and other secreted proteases. Notably, OV19’s collagenolytic performance was markedly higher than that of the other pathogenic reference strains tested, including *V. vulnificus* and *V. parahaemolyticus*, both well-documented collagenase producers [34], as well as other Harveyi clade members (Figure 3B). These findings are consistent with the severe tissue lysis observed in the infected octopus and support a role for OV19’s collagenolytic arsenal in virulence.

We next evaluated motility, another trait frequently linked to *Vibrio* pathogenicity [35]. Motility assays conducted under controlled conditions revealed that OV19 displayed pronounced swarming motility within a 3 h observation period (Figure 3B. In *Vibrio*, motility confers ecological advantages in nutrient-limited aquatic environments, besides playing a critical role in host colonization, tissue invasion, and immune evasion [35].

The combination of potent collagenolytic activity and strong motility suggests that OV19 possesses multiple virulence-associated traits that could act synergistically during infection. In the case of the diseased octopus, these features may have enabled the bacterium to promote rapid tissue destruction, bacterial dissemination, and the fulminant septicemia observed. These results support the positioning of OV19 closer to an actively invasive pathogen than to an opportunistic colonizer.

Given these findings, further genomic investigations, particularly whole-genome sequencing, might help unveil the genetic basis of OV19’s enzymatic repertoire, which is of potential interest for subsequent biotechnological exploitation (e.g., production of recombinant enzymes) and its pathogenic potential, though the latter should be experimentally assessed. Moreover, WGS might allow for a more precise identification of the strain, although it should be pointed out once again that the taxonomic classification of vibrios is challenging, mostly due to their high genomic plasticity and recombination rates [8,33].

## 3. Materials and Methods

### 3.1. Specimens’ Collection, Bacterial Isolation and Cultivation

Skin and muscle from a wild octopus (*Octopus vulgaris*) were sampled by cutting with a scalpel, weighed, homogenated in sterile 2% NaCl, and roughly cleared by sedimentation on ice for 20 min. Haemolymph was sampled from the heart using a sterile syringe. Samples were serially diluted in sterile 2% NaCl, then plated on Marine Agar (Condalab, Madrid, Spain) and mTCBS-Agar (modified TCBS) (Condalab, Madrid, Spain) as described in [26].

Single colonies were selected (also based on colony phenotype), isolated and cultivated in Marine Broth (Condalab, Madrid, Spain).

*Vibrio* type strains were from DSMZ (Braunschweig, Germany). *V. parahaemolyticus* (DSM10027), *V. vulnificus* (DSM10143), *V. alginolyticus* (DSM2171), *V. owensii* (DSM2165), *V. campbellii* (DSM19270), *V. harveyi* (DSM19623), and *V. splendidus* (DSM19640) were cultivated in Marine Broth.

### 3.2. Molecular and Bioinformatic Analyses

DNA for PCR amplifications was prepared from single colonies of isolates grown on Marine Agar using the procedure of fast lysis described in [36]. PCR for *Vibrio* screening were carried out using the *Vibrio*-specific primers (rpoD_66M13F/rpoD_1592R) described in [26].

Primers employed to amplify genomic *loci* from *Vibrio* for MLSA are described in [28]. The universal primers pair 27F/1492R [37] was used to amplify the *16S* rDNA. Amplicons were purified using the Gfx PCR DNA Gel Purification Kit (GE Healthcare, Chicago, IL, USA) and sequenced by the Sanger method by Macrogen Europe (*Milano,* Italy). Following reads trimming, the obtained sequences underwent concatenation [28] and phylogenetic cluster analyses using MEGA11: Molecular Evolutionary Genetics Analysis software, version 11.0, Pennsylvania State University, USA (https://www.megasoftware.net).

The multilocus sequence analysis (MLSA) approach was carried out by concatenating conserved genetic markers for each strain. Concatenated sequences were compared with reference databases to identify the closest related taxa, and a representative dataset was assembled including related species and an appropriate outgroup. Phylogenetic trees were reconstructed using multiple inference methods to ensure consistency and robustness of the resulting topology [28,29,30].

### 3.3. Screening of Protease-Producing Bacteria

The trypsin-like activity of the supernatant of different isolated *Vibrio* strains was assessed using the BOC-Gln-Ala-Arg-AMC- (PeptaNova GmbH, Keplerstr, Andhausen, Germany) specific peptides, at concentrations of 0.025 mM [38]. The analysis was carried out in TES buffer, pH 7.4. Unless otherwise stated, reactions were set up in a total volume of 200 μL in microtiter wells and incubated at 37 °C. Fluorescence was measured for 30 min using wavelengths of 355 nm for excitation and 460 nm for emission, in a Biotek Synergy HT microplate reader (BioTek, Winooski, VT, USA). Enzyme-free reactions were used as a negative control and background fluorescence was subtracted from each value. All experiments were conducted in triplicate.

### 3.4. SDS-PAGE and Zymography

Supernatants from liquid cultures were analyzed in gelatin zymography. Each sample was separated in 7.5% polyacrylamide gel containing 1 mg/mL bovine gelatin, under non-reducing conditions [39]. After electrophoresis, gelatin zymographies were incubated for 24 h at 37 °C in activation buffer containing 2 mM CaCl_2_, 50 mM Tris-HCl buffer, 1.5% Triton X-100, and 0.02% NaN_3_, pH 7.4. After overnight incubation at 37 °C, gel was stained using Coomassie Brilliant Blue G-250. (Sigma Aldrich, Milan, Italy)

### 3.5. Proteases and Collagenase Activity

Collagenolityc activity was also evaluated by the enzymatic assay of collagenase using Carbobenzoxy-Gly-Pro-Gly-Gly-Pro-Ala-OH as substrate [22,40]. The synthetic peptide Carbobenzoxy-Gly-Pro-GlyGly-Pro-Ala-OH is specifically hydrolyzed by collagenase in two fragments: the Carbobenzoxy-GlyPro-Gly and Gly-Pro-Ala, which reacts with the ninhydrin and can be quantified by a spectroscopic. In this assay 1 U catalyzes the hydrolysis of 1 micromol of Gly-Pro-Ala from Z-GlyPro-Ala (Fluka, Milan, Italy) in 1 min at pH 7.4 and 37 °C. To investigate the proteases activities, we measured them using a modification of the digestion method in which the enzymes were incubated for 5 h with casein (Sigma-Aldrich, St. Louis, MO, USA) at 37 °C. The casein digestion was determined using the colorimetric ninhydrin process. The amino acids released are expressed as micromoles leucine per milligram dry weight of enzyme. One unit equals one micromole of leucine equivalents released from collagen in 5 h at 37 °C, pH 7.5, under the specified conditions.

### 3.6. Collagen Degradation Test

Bacteria were grown in Marine Broth at 28 °C with orbital shaking overnight, diluted 1:100 in the same medium, and grown up to 1 OD_600_. Exponentially growing cells were diluted 1:5000 in sterile sea water in the presence of 100 mg of insoluble collagen from bovine Achilles tendon (Sigma Aldrich, Milan, Italy) [41], and grown overnight with shaking at 28 °C. The undigested collagen was recovered by centrifugation (4000× *g* for 5′), washed twice in ddH_2_O, then with 90% ethanol, and then air-dried and weighted. Thereafter, the percentage of digestion was calculated, considering 100 mg as the initial amount.

### 3.7. Swarming Motility Test

The motility was assessed following the procedure described in [35] with some modifications. Briefly, 8 mL of Marine Agar (MB + 0.5% agarose) were poured in a 60 mm Petri dish, and a micro-well was formed in the center using a pipet plastic tip. 10 μL of cells grown in MB at 30 °C up to 1.5 OD_600_ were put in the micro-well, kept at 25 °C and monitored over time.

## 4. Conclusions

This study identified the OV19 isolate as a *Vibrio* strain, member of the Harveyi clade, closely related to *V. owensii* but likely distinct from any WGS-sequenced strain present in databases. From an evolutionary standpoint, OV19 exemplifies the genetic variability characteristic of the *Vibrio* genus, in which high recombination rate and genome plasticity drive the emergence of highly adapted and pathogenic phenotypes, which make taxonomic classification challenging.

Comparative biochemical analyses demonstrated that OV19 possesses high collagenolytic and proteolytic activities capable of inducing extensive tissue lysis through efficient degradation of structural proteins—including collagen—thereby facilitating tissue invasion and damage. However, it is worth noting that no experiments specifically designed to demonstrate the actual pathogenicity or virulence of the OV19 strain were conducted in this study. Consequently, its true pathogenic role remains unproven and would require targeted investigations to be confirmed.

Collectively, our findings further highlight the variability of vibrios and the potential of emergence of novel strains harboring unique features, including the production of molecules with high potential of biotechnological exploitation.

## Data Availability

The original contributions presented in this study are included in the article. Further inquiries can be directed to the corresponding author(s).
